# Predicting high recombinant protein producer strains of *Pichia pastoris* Mut^S^ using the oxygen transfer rate as an indicator of metabolic burden

**DOI:** 10.1038/s41598-022-15086-w

**Published:** 2022-07-02

**Authors:** David Wollborn, Lara Pauline Munkler, Rebekka Horstmann, Andrea Germer, Lars Mathias Blank, Jochen Büchs

**Affiliations:** 1 Chair of Biochemical Engineering (AVT.BioVT), RWTH Aachen University, 52074 Aachen, Germany; 2 iAMB - Institute of Applied Microbiology, RWTH Aachen University, 52074 Aachen, Germany

**Keywords:** Expression systems, Industrial microbiology, Sequencing, Chemical engineering

## Abstract

The methylotrophic yeast *Pichia pastoris* (*Komagataella phaffii*) is a widely used host for recombinant protein production. In this study, a clonal library of *P. pastoris* Mut^S^ strains (^S^ indicates slow methanol utilization) was screened for high green fluorescent protein (GFP) production. The expression cassette was under the control of the methanol inducible *AOX* promoter. The growth behavior was online-monitored in 48-well and 96-well microtiter plates by measuring the oxygen transfer rate (OTR). By comparing the different GFP producing strains, a correlation was established between the slope of the cumulative oxygen transfer during the methanol metabolization phase and the strain’s production performance. The correlation corresponds to metabolic burden during methanol induction. The findings were validated using a pre-selected strain library (7 strains) of high, medium, and low GFP producers. For those strains, the gene copy number was determined via Whole Genome Sequencing. The results were consistent with the described OTR correlation. Additionally, a larger clone library (45 strains) was tested to validate the applicability of the proposed method. The results from this study suggest that the cumulative oxygen transfer can be used as a screening criterion for protein production performance that allows for a simple primary screening process, facilitating the pre-selection of high producing strains.

## Introduction

The biotechnological production of recombinant proteins is an important industrial sector addressing applications such as the food and feed industry, industrial enzymes or biopharmaceuticals^1–3^. A well-established microbial protein production host is the methylotrophic yeast *Pichia pastoris* (reclassified as *Komagataella phaffii*^4^). The production of new target proteins entails genetic strain engineering that normally results in large, heterogenic clone libraries^5^. Primary screening campaigns are performed to find strain variants that produce high levels of the desired protein. Strain selection has a major impact on the economic feasibility of the final production process, thus, it is important to test all generated strains^6^. However, testing large clonal libraries is not an easy endeavor. Product quantification requires additional efforts, as the activity or the amount of protein produced needs to be quantified by additional analytical methods. As a result, the size of the generated clone library and the screening effort required, become the cost and time intensive bottleneck within the development of new bioprocesses^6^.

It is well known that the production of large amounts of recombinant proteins heavily affects the cell’s metabolism. The host cell’s energy and metabolite resources are limited and must be divided between growth-associated processes and protein synthesis^7^. Excessive protein production can be reflected in the growth behavior, as protein synthesis outweighs growth-associated processes^8–10^. It is reported that, when a decrease in the growth rate is detected, an increase of the recombinant protein content is observed^11^. This phenomenon is well-known for different production hosts (e.g., *Escherichia coli* and *P. pastoris*) and described as metabolic burden, metabolic load or metabolic drain^12^*.*

When metabolic burden becomes apparent in the metabolism of a strain, the effect can be exploited for the screening process and used as an evaluation criterion. Technologies such as the BioLector^13^ or the Respiration Activity Monitoring (RAMOS)^14,15^ system allow to online monitor the metabolic state of individual strains during the screening process.

For *E. coli* it has already been successfully demonstrated that differences in productivity and growth behavior are visible in the course of the online-monitored oxygen transfer rate (OTR)^16^. In *E. coli* cultivations, the OTR is such a sensitive parameter that even the metabolic burden effects, caused by the exchange of a single amino acid in the protein of interest (POI), can be detected in the OTR^17^.

For *E.* coli it has also been demonstrated that the obtained course of the scattered light signal in BioLector cultivations can be used to predict the production performance^18^. Additionally, the scattered light signal can be related to the physiological state of an *E. coli* strain and can be used to create a score value for each individual clone, representing the performance in terms of recombinant protein production^19^. Thus, it can be concluded that it is possible to estimate the amount of protein produced from the observed metabolic burden, based only on online measured OTR or scattered light data. This has the advantage that other common methods for recombinant protein detection do not need to be applied. These include endpoint measurements via assays such as ELISA or SDS-PAGE, and also fusion tags. Commonly used fusion tags include His-tags^20^or Strep-tags^21^. These tags enable an easy and specific protein recovery at the end of the fermentation. Another common practice is the fusion of the protein of interest to fluorescent proteins or molecules that allow the online measurement of the protein’s formation. Some examples are fusions to GFP^22^ or fluorescent proteins containing flavin mononucleotides-based fluorescent proteins (FbFPs)^23^. Additionally, tryptophan (W) tags^24^ can be measured online. The disadvantage of protein fusions is that the native structure of the protein of interest can be affected. Moreover, these tags often cannot remain fused to the protein of interest and need to be removed after successful production. Lastly, the production of the fusion proteins themselves is a burden to the cells. One approach to minimize the burden caused by the co-expression of a large fusion protein is the use of a split-GFP variant^25,26^. This reduces the size of the fusion partner, because the protein of interest is fused with a 16-amino-acid residues tag that complements a truncated GFP. For endpoint quantification, the protein of interest, including the amino-acid tag, is mixed with a previously produced truncated GFP. The result is a complemented GFP that restores the fluorescent signal. Nevertheless, using the GFP amino-acid tag does not entirely avoid additional metabolic burden.

In this study, we demonstrate that the phenomenon of metabolic burden is not only applicable for *E. coli* but also for the expression host *P. pastoris* Mut^S^. In the Mut^S^ phenotype, the alcohol oxidase 1 gene (*AOX1)* is deleted, leading to a reduced methanol utilization rate compared to the wild type strain. Only the alcohol oxidase 2 is produced by the cells, causing a slow (“s”) methanol metabolization. Nevertheless, methanol can still be used for anabolic and catabolic cellular processes^27^. We deploy the frequently used alcohol oxidase (*AOX1*) promoter to control GFP production. This promoter is derived from the methanol utilization pathway (Mut) and induced in the presence of methanol^28^. To simplify the screening, an auto-induction approach was used^29–31^. This means, that 2% v/v methanol was added right from the beginning of the cultivation, and the cells shift towards methanol consumption upon depletion of glycerol. Together with this shift, the *AOX1* promoter is induced and GFP production is initiated. This method differs from regular methanol induction procedures, where methanol is added in pulses after depletion of the main carbon source. Cultivations were conducted in an in-house built combined µRAMOS/BioLector system that allows online-monitoring of the OTR, scattered light, and GFP fluorescence at 48-well plate microtiter plate (MTP) scale^32,33^. The RAMOS technology was also extended and applied to 96-deepwell microtiter plates^34^. 96-well MTP are the standard format for screening campaigns, because they allow the parallelization of cultivations at reasonable number and costs^35,36^*.* Using the course of the OTR over time as strain selection criterion allows to circumvent the necessity to take offline samples for protein quantification during primary screening. The method is also independent of protein purification tags or labels. As a result, it simplifies and accelerates the overall primary screening process.

## Results and discussion

### The oxygen transfer rate at MTP scale can be separated into 4 phases

**Figure 1:**
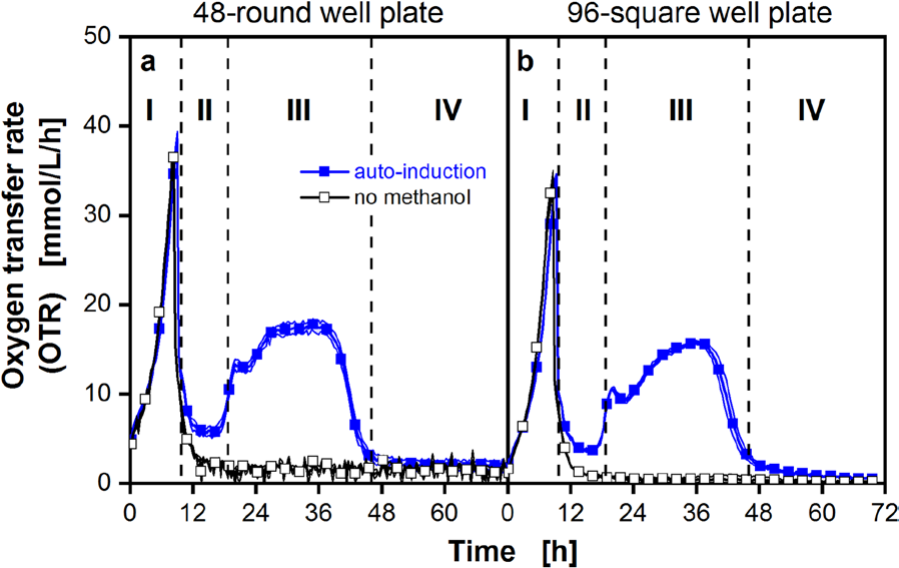
Cultivation of *P. pastoris* Mut^S^ reference strain in two types of microtiter plates with simultaneous online monitoring of the oxygen transfer rate (OTR). Cultivation was carried out (**a**) in a 48-round well microtiter plate (MTP), 0.8 mL filling volume per well, operated at 1000 rpm and 3 mm shaking diameter in a µRAMOS/BioLector^32^ combination device and (**b**) in a 96-square well MTP, 0.6 mL filling volume per well, operated at 350 rpm and 50 mm shaking diameter in a µTOM device^34^. The OTR at both MTP scales is depicted without methanol addition (black line), and with 2% (v/v) of methanol added to the medium at the beginning (t = 0 h) of the cultivation to obtain auto-induction conditions (dark blue line). The data was obtained in N = 2–6 replicates. Shadows symbolize the standard deviation of cultivations for N ≥ 3. In both panels, for clarity, only every 8th data point is displayed. The OTR course for the cultures with auto-induction can be divided into 4 phases (I to IV) at both MTP scales, separated by vertical black dashed lines: Phase I: growth on glycerol until depletion, phase II: methanol adaption, phase III: methanol consumption phase and phase IV, methanol is depleted. Cultivation conditions: mineral Syn6 medium with 10 g/L glycerol and 200 mM MES buffer (pH = 6.0). Cells with an initial optical density (OD_600nm_) of 0.9 were cultured at 30 °C.

For all cultivations in this study, an auto-induction screening method was chosen. Briefly, 2% v/v methanol was added to mineral Syn6-MES medium with 10 g/L glycerol at the beginning of the cultivation. The corresponding OTR over time at 48-round well and 96-square well MTP scale is shown in Fig. 1a and b, respectively. A *P. pastoris* Mut^S^ strain, without a GFP expression cassette, was cultivated in the auto-induction medium, at 48-round well and 96-square well MTP scale. From Fig. 1 it becomes apparent that the course of the OTR can be divided into 4 phases (I to IV) at both MTP scales. Phase I is growth on glycerol until depletion. The exponential growth is reflected in the exponential increase of the OTR signal, until the OTR reaches a maximum of about 38 mmol/L/h (Fig. 1a) and 35 mmol/L/h (Fig. 1b), respectively, after 9 h of cultivations. This peak marks the end of cultivation phase I and the signal sharply declines as no more glycerol is available. The correlation of OTR and carbon source depletion has been validated several times for different host strains, using offline analysis^37,38^. Importantly, cultures cultivated under auto-induction conditions do not drop to an OTR of 0 mmol/L/h. They maintain a breathing activity between 4 and 6 mmol/L/h, whereas the breathing activity of the methanol free cultivations stops (Fig. 1a and b, black hollow squares). This growth behavior is observed at both MTP scales (Fig. 1). Based on this observation, this growth phase is referred to as phase II: methanol adaption. During this phase, the cells undergo a change in their metabolism towards the methanol utilization (MUT) pathway^27,39^.

Phase III is defined as the methanol consumption phase. Once the cells switch towards the MUT pathway, methanol can be slowly consumed and as a result, the OTR rises again, forming a plateau between 15 and 17 mmol/L/h (Fig. 1). As this plateau remains at a rather constant level during phase III, it can be assumed that the cells metabolize methanol at their maximum capacity. This assumption is supported by the used Mut^S^ phenotype, were the alcohol oxidase 1 gene (*AOX1*) has been deleted. As a result, the cells metabolize methanol at a reduced rate^27,40^, and with methanol present in excess, the consumption is at its maximum and therefore constant (constant OTR plateau)^41^.

In phase IV, the OTR drops slowly, until it reaches 0 mmol/L/h. That indicates the end of breathing activity and, therefore, methanol depletion. To prove that the observed OTR course in phases II and III can be traced back to methanol consumption, a negative control was included, were no methanol was added (Fig. 1, black hollow squares). When methanol was present at both MTP scales, a plateau was formed and the overall course was highly comparable throughout the different phases.

**Figure 2:**
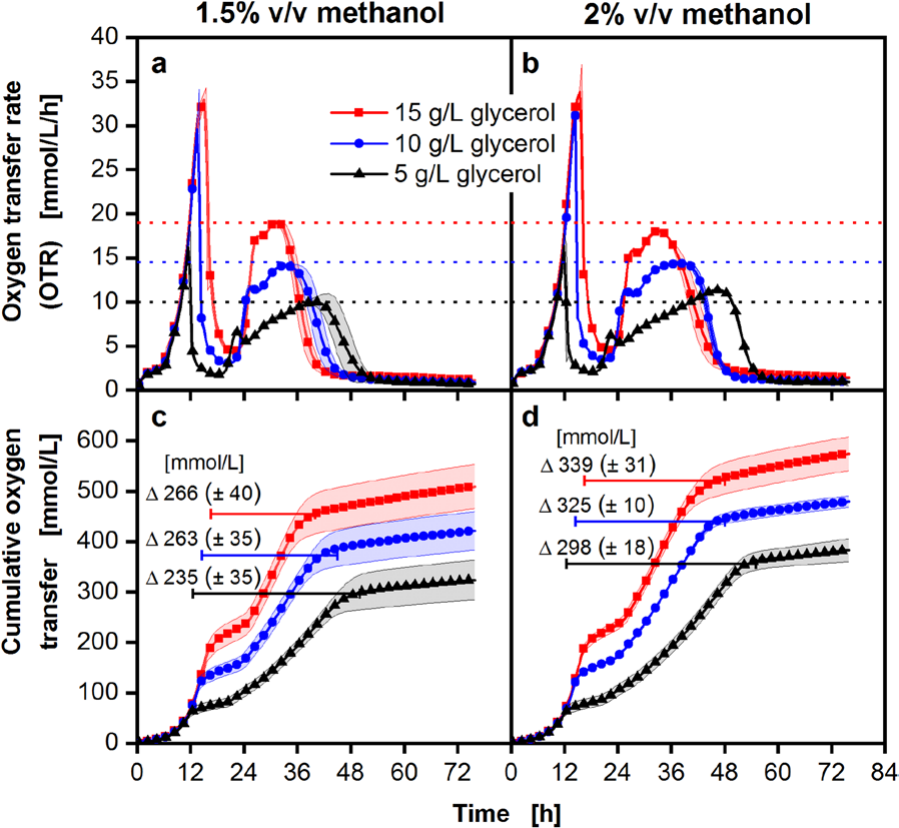
Cultivation of *P. pastoris* Mut^S^ reference strain at different glycerol and methanol concentrations in microtiter plates with simultaneous online monitoring of the oxygen transfer rate (OTR). Cultivation was carried out in a 96-square well microtiter plate, 0.6 mL filling volume per well, 350 rpm and 50 mm shaking diameter, in a µTOM device^34^. The OTR is depicted for cultivations with 5, 10 and 15 g/L glycerol, using auto-induction conditions, with (**a**) 1.5% v/v methanol and (**b**) 2% v/v methanol added at the beginning of the cultivation. The cumulative oxygen transfer for the 1.5% v/v conditions is depicted in (**c**) and for the 2% v/v methanol conditions in (**d**). In all panels, for clarity, only every 4th data point is displayed. Shadows symbolize the standard deviation of cultivation replicates (N = 3). The dotted horizontal lines in (**a**) and (**b**) mark the maximum OTR value of (**a**) for the tested conditions during phase III: methanol consumption phase. Additionally, the volumetric oxygen amount consumed during growth on methanol is displayed for the different conditions in (**c**) and (**d**). The values were obtained by calculating the difference of the cumulative oxygen transfer between the end of phase I and the beginning of phase IV. The position and length of the horizontal bars in (**c**) and (**d**) mark the duration of phase II and III. The obtained value corresponds to the total volumetric amount of oxygen consumed in phases II and III (see Fig. 1). Cultivation conditions: mineral Syn6 medium with 200 mM MES buffer (pH = 6.0). Cells with an initial optical density (OD_600nm_) of 0.2 were cultured at 30 °C.

To show how the height and length of the observed methanol utilization phase (phase III) is influenced, the amount of glycerol and methanol were varied (Fig. 2). These experiments were performed at 96-square well MTP scale. For all cultivations, the same Syn6-MES auto-induction medium was used. The phases I to IV have not been marked as the length of the different phases differ between the tested conditions. A variation in the glycerol concentration (5, 10, and 15 g/L) results in the formation of more biomass during phase I and becomes apparent in higher and longer OTR peaks for the different conditions (Fig. 2a and b). The methanol utilization rate for Mut^S^ strains seems to be limited to a maximum rate, represented by a plateau in phase III. When a higher biomass is formed, prior to methanol consumption, a higher OTR plateau is observed during phase III. For the 5 g/L glycerol conditions, the cells reach a maximum OTR during phase III between 10 and 11.5 mmol/L/h. While the 10 g/L glycerol conditions reach an OTR value of 14 mmol/L/h for the plateau in phase III. As expected, the highest OTR value during phase III is reached for the highest glycerol concentration of 15 g/L, with a value of 18–19 mmol/L/h. Thus, it can be interpreted, that the height of the observed plateau is mostly influenced by the amount of used glycerol, which result in different amounts of biomass, and not by the methanol concentration used (1.5% v/v in Fig. 2a vs. 2% v/v in Fig. 2b).

In contrast, higher methanol concentrations result in a longer phase III. To quantify this effect, methanol evaporation needs to be taken into account. The length of phase III for the different conditions is summarized in Supplementary Table 1 (3rd column). Furthermore, methanol evaporation under the used cultivation conditions was determined for 96-square well plates via HPLC measurements over time (Supplementary Fig. 1). The obtained data was used to fit an equation for methanol evaporation (Supplementary Eq. 1). This data allows to estimate the amount of methanol that the cells theoretically had for consumption during cultivation (Supplementary Table 1, 4th column). These theoretical methanol concentrations are transformed into a volumetric oxygen amount (Supplementary Table 1, 5th column) that would be necessary to completely oxidize methanol to carbon dioxide and water. For this purpose, a simplified assumption of combustion was made (Supplementary Eq. 2).

The experimentally measured oxygen consumption for the different conditions tested is highlighted in Fig. 2c and d. The individual length of phase III is marked, together with the consumed oxygen amount (see also Supplementary Table 1, 3rd, and 6th column). This procedure underestimates the amount of oxygen needed, as the obtained experimental data is about 16% higher than the amount of theoretically required oxygen (Supplementary Table 1, last column). A possible explanation is that metabolic activity during phase II is also taken into account. During this phase, it is conceivable that oxygen is also needed for other metabolic processes besides methanol utilization. It must also be considered, that the evaporation rate is overestimated, as methanol consumption and evaporation happen simultaneously. A lowered methanol concentration in the medium also lowers the evaporation rate. As a result, the amount of methanol available to the cells is higher, leading to a higher oxygen demand. Additionally, assuming pure combustion is a rather simple approach and can only be regarded as an approximation. However, considering the errors in media preparation and the OTR measurement, the obtained data indicates a closed mass balance for methanol during phase II and III. The intention of this study is to emphasize that a higher initial methanol concentration results in an extended phase III, but not in a higher OTR plateau. In contrary, different initial glycerol concentrations result in different biomass concentrations, which affect the length and height of phase III. Nevertheless, the cumulative oxygen consumption under consideration of evaporation is very similar for the conditions tested using 1.5% v/v and 2% v/v methanol (Supplementary Table 1, 6th column).

### The course of the OTR over time indicates metabolic burden

**Figure 3:**
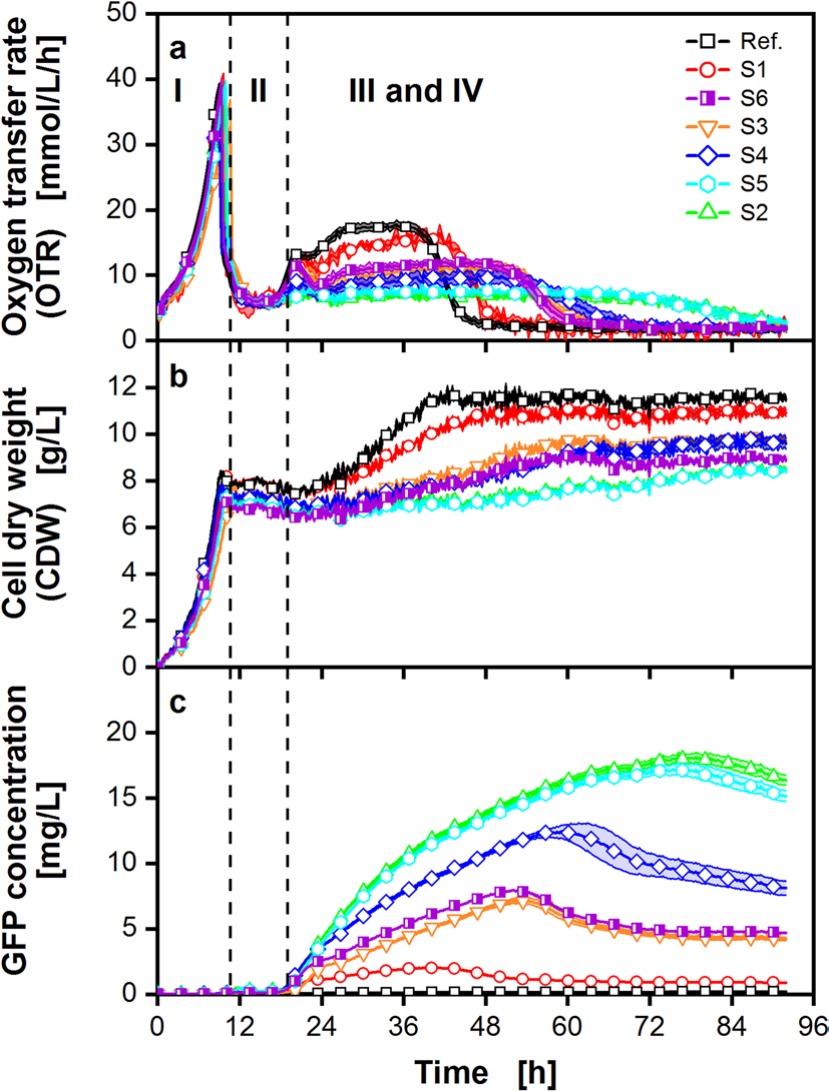
Cultivation of a small clone library of GFP-secreting *P. pastoris* Mut^S^ strains in a 48-round well microtiter plate with online monitoring of the oxygen transfer rate (OTR), scattered light and GFP fluorescence, measured in a µRAMOS/BioLector combination device^32^. Results are depicted for a cultivation with 10 g/L glycerol and 2% (v/v) of methanol added at the beginning of the cultivation, to obtain auto-induction conditions. (**a**) OTR course for 6 GFP-secreting strains and a no GFP-producing reference strain. For clarity, only every 12th data point is displayed. (**b**) Biomass by backscatter (I-I0) (every 20th data point displayed), (**c**) GFP fluorescence (every 20th data point displayed). The three cultivation phases (I-III) are identified by the OTR curves and are separated by vertical black dashed lines (see Fig. 1). Cells with an initial optical density (OD_600nm_) of 0.9 were cultured at 30 °C in mineral Syn6 medium with 200 mM MES buffer (pH = 6.0). Cultivation conditions: 0.8 mL filling volume per well, 1000 rpm shaking frequency, 3 mm shaking diameter. Each cultivation was performed in triplicates. Light shadow depicts the standard deviation. In some cases, the standard deviation is so small that the error is not visible.

A pre-selected library of seven GFP-secreting *P. pastoris* Mut^S^ strains, high (S5, S2), medium (S6, S4, S3), and low (S1) GFP producers, was screened under auto-induction conditions using an in-house built BioLector device that allows to online-monitor the OTR, backscatter, and GFP fluorescence in every individual well of a 48-round well plate^32,33^. The obtained results are depicted in Fig. 3. Figure 3a demonstrates that the 4 previously mentioned phases are again visible in the OTR signal. All tested strains perform identical in phase I, as they were inoculated to the same initial OD_600_ and with the same amount of glycerol. The OTR increased to a maximum of 36–38 mmol/L/h after 9.3 h. After the peak, the OTR dropped sharply to 6 mmol/L/h, indicating glycerol depletion. Phase II was also identical for all tested strains, however, the OTR course differed once the cells entered the methanol adaption and metabolization phase after 19 h (phase III). It was observed that the length and height of the OTR at phase III strongly differed between the tested strains. After 23 h, the OTR of all strains plateaued at different heights. Meanwhile, the reference strain (Ref.; black squares) and S1 (red circles) reached a high OTR level of 17 mmol/L/h and 15 mmol/L/h, respectively. A low OTR of 7 mmol/L/h was detected for the clones S5 (cyan hexagon) and S2 (green triangles).

These differences result in strain-specific durations of phase III, and phase IV is reached at different time points. This observation was unexpected, as the experimental data in Fig. 2 shows that the height and length of the OTR plateau in phase III should be the same, if the cultivation conditions are identical in terms of glycerol and methanol availability. Therefore, there must be strain specific differences affecting the MUT pathway. Differences are also visible in the online monitored scattered light data that was converted to a cell dry weight (CDW) value by a correlation (Fig. 3b). During the first 12 h, CDW increased exponentially for all tested strains to values between 7.2 and 8 g/L. The signal is highly comparable until 24 h. Once the strains enter phase III (19 h), a strain-specific increase in CDW is observed, which varies in its slope and lasts until methanol is depleted. The strongest increase is visible for the Ref. and S1 strain (Fig. 3b). This steady increase of CDW seems to correlate with a high OTR plateau (17.5 mmol/L/h for the Ref. and 15.5 mmol/L/h for the S1 strain). Thus, the higher OTR plateau for the Ref. and S1 strains can be correlated to the higher increase of biomass, compared to the other tested strains. Generally, the biomass remained constant after the strains entered phase IV.

In Fig. 3c, for all investigated strains, the GFP signal started increasing at 19 h. This coincided with the rise of the OTR in phase III of the cultivation. The clones S5 and S2 achieved the highest GFP concentration of 18 and 17 mg/L. While the S1 strain showed the lowest GFP concentration of 2 mg/L. The obtained GFP concentrations for the high producing strains (S2 and S5) were highly comparable to literature, where GFP concentrations between 0.5 mg/L in batch mode and 30 mg/L in fed-batch operation mode were reported for the closely related methylotrophic yeast *Hansenula polymorpha*^42^. Furthermore, it was shown for *E. coli* cultivations, that metabolic burden can already be caused by the production of a cellulase with an activity between 200 and 800 µU/mL^19^. This corresponds to a cellulase concentration of approximately 13–53 mg/L when a specific activity of 15 U/mg is assumed^43^. Moreover, it was also shown for *E. coli*, that the total amount of formed protein is not crucial, as metabolic burden can already be caused by single amino acid exchanges in the target protein^17^. Therefore, the observed differences between the strains, in the scattered light and the OTR, are most likely caused by a deregulation of the metabolism. However, elucidating the underlying principles that are involved are beyond the scope of this study.

When comparing the three online monitored process parameters, a correlation between high GFP concentration with a low increase of CDW and a low OTR plateau becomes apparent. A similar correlation has already been described for *E. coli* cultivations by Rahmen et al*.*^17,44^. The low OTR plateau additionally results in an extended duration of phase III. These findings highlight that a high recombinant protein (GFP) titer usually results in less biomass formation. This is further supported by the observation that the differences in the growth rate only appear during phase III. As the methanol inducible p*AOX* promoter is used to control GFP expression, the cells are fully induced during phase III. This is also visible in the monitored GFP signal (Fig. 3c). However, it has already been shown for *E. coli* that the recombinant protein production performance can be estimated from the progression of the scattered light signal (biomass formation) during the course of a cultivation^18,19^. Differences were already visible when a cellulase CelA2 was produced in quantities of approximately 13–53 mg/L (200–800 µU/mL respectively, with a specific activity of 15 U/mg)^19,43^.Furthermore, the presence of a heterologous gene impacts the cells’ physiology substantially. Thereby, the amount of protein being produced is not as important as what kind of protein. For example, it was shown for *E. coli* that even individual amino acid exchanges cause metabolic burden of varying intensity, which is visible in the scattered light^17^. The observed differences in growth and production behavior can be explained by the concept of metabolic burden^11,12^. The higher the metabolic burden, the higher the protein production of the respective clone. In this case, a maximum GFP concentration of 18 mg/L seems sufficient to cause metabolic burden. Accordingly, both, the scattered light (resp. CDW) signal and the OTR, can be used to evaluate the metabolic burden of the production host *P. pastoris*. Differences in growth and respiration activity could also arise from changes in the methanol metabolization pathway, due to random integration of the heterologous gene or mutations. Nevertheless, metabolic burden is likely caused by heterologous protein production, as supported by the results obtained for the strains S3 and S6. Both strains carry 2 copies of the GFP cassette, which are integrated in different loci (Supplementary Fig. 4). Despite the variable integration site, they perform almost identical, according to OTR and growth (Fig. 3). Additionally, the gene copy number of the different strains (S1 to S6) reflects the obtained rating as well, supporting the metabolic burden concept. Thus, online biomass determination methods (e.g., BioLector (Beckman Coulter), the Cell Growth Quantifier (Aquila biolabs), Growth Profiler (Enzyscreen) or the Bioscreen C (Oy Growth Curves)), as well as the OTR can be online monitored and used for strain ranking. For *E. coli* cultivations, the BioLector technology was already successfully applied for the prediction of recombinant protein production performance^18,19^.

**Figure 4:**
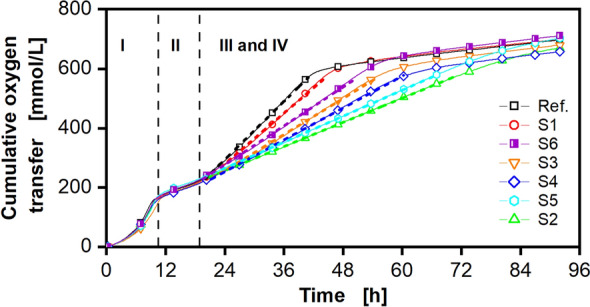
Cumulative oxygen transfer data of a clone library with GFP-secreting *P. pastoris* Mut^S^ strains cultivated in a 48-round well microtiter plate. The integral of the OTR (cumulative oxygen transfer) of the cultures shown in Fig. 3 is depicted. For clarity, only every 20th data point is displayed. The four cultivation phases (I-IV) are indicated by vertical dashed lines as in the previously shown data set (Fig. 3). The slope of the cumulative oxygen transfer was determined by a linear fit of phase III (drawn as dashed line in the corresponding color). The slope values are summarized in Supplementary Table 2.

**Figure 5:**
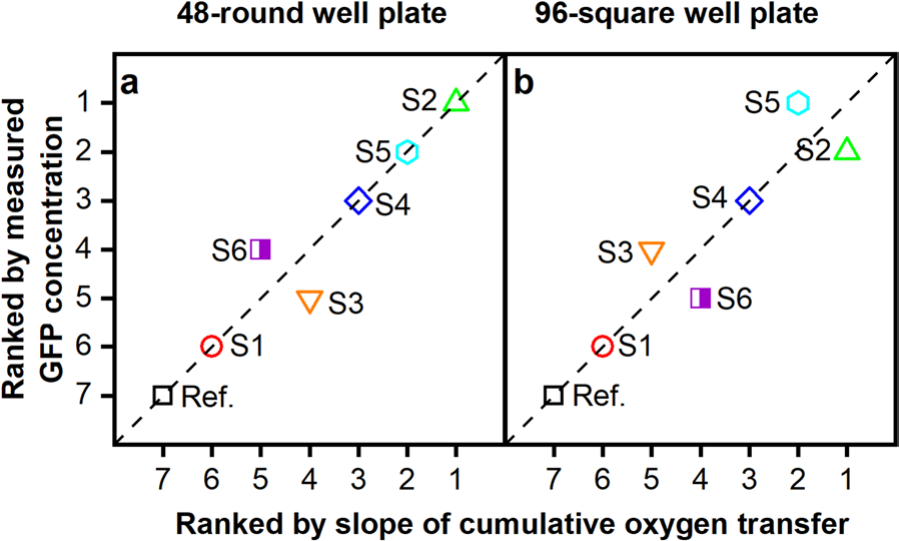
Parity plot of the ranking of a clone library with GFP secreting *P. pastoris* Mut^S^ strains. The seven different strains were ranked by the GFP concentration measured at the end of the cultivation (y-axis). The highest GFP fluorescence results in rank 1 (best) and gradually decreases according to the GFP value obtained. On the x-axis, the slope of the cumulative oxygen transfer was used as ranking criterion (see Supplementary Table 2). Here, the lowest slope is ranked as 1 (best). If the data points lie on the angular bisector, the ranking result was identical for both methods. The results for a cultivation in 48-round well plates (Fig. 3) is shown in (**a**) and for the cultivation in 96-square well plates (Supplementary Figs. 2 and 3), respectively, in (**b**).

To further analyze and quantify the correlation between OTR and production performance, the cumulative oxygen transfer was calculated from the measured OTR data (Fig. 4). All strains were cultivated under identical conditions. As a result, the final total amount of consumed oxygen is nearly the same for all tested strains (660–710 mmol/L), notwithstanding different kinetics. The plot nicely illustrates how the different strains differ in their oxygen consumption rate during phase III. The consumption rate is equal to the slope of the obtained data during phase III. It was determined via linear regression and is depicted in Fig. 4 as a dashed line for each individual strain. The same procedure was followed for a screening at 96-square well MTP scale (see Supplementary Figs. 2 and 3). The quantitative values of the slopes from Fig. 4 and Supplementary Fig. 3 are presented in Supplementary Table 2. The observed differences in the oxygen transfer rate during phase III suggest that the slope of the cumulative oxygen transfer can be used as ranking criterion. The evaluation method based on OTR is particularly advantageous, because the BioLector technology cannot be applied to the standard 96-square well MTP scale, as these plates do not have a transparent bottom. Only the OTR was online monitored and can be used to evaluate the strain performance. For the screening at 96-square well scale, the same pre-selected clone bank was cultivated. The obtained courses of the OTR over time are in agreement with the results at 48-round well MTP scale (Supplementary Figs. 2 and 3). In Fig. 5, the ranking results at both scales are summarized. There, the lowest slope (see Supplementary Table 2) was ranked as 1 (best) and the highest slope as 7 (worst).

### Using the OTR as ranking criterion for strain performance leads to a similar ranking as end-point protein measurements

The screening results of the pre-selected library of seven GFP-secreting *P. pastoris* Mut^S^ strains at 48-round well and 96-square well scale were compared. The data is summarized in a parity plot (Fig. 5). Here, the ranking result from the cumulative oxygen transfer on the x-axis is plotted against the ranking result that derived from end-point offline GFP measurements. The GFP concentration was calculated from a GFP standard of known concentration to enable the conversion of the fluorescence signal from the BioLector to a GFP concentration. This method takes only correctly folded and functional GFP into account.

Figure 5a shows that at 48-round well plate scale, the ranking for the tested strains is very similar. The data points match the angular bisector, meaning, that the ranking result was identical, regardless of whether the measured GFP concentration or the slope of the cumulative oxygen transfer was used as ranking criterion. Only strains S3 and S6 show a reversed ranking. The results for the screening of the same strains at 96-square well scale (Fig. 5b) also show that the obtained screening results are close to the angular bisector. Besides the already described discrepancy of the ranking for strains S3 and S6, there is also a change in the ranking of strains S2 and S5. To further investigate the ranking results, the strains were sent for Whole Genome Sequencing (WGS). The sequencing data allows to determine the number of integrated GFP expression cassettes. The obtained copy numbers were in accordance with the observed differences in strain performance (Table 1). Additionally, from the WGS results, a possible explanation for the observed changes in ranking can be given. The strains S3 and S6 contain 2 copies of the GFP expression cassette and the strains S2 and S5 4 copies respectively. Therefore, due to the same gene copy number, it is likely that strains S3, S6 and S2, S5 perform very similar in terms of heterologous protein production, due to the same gene copy number. This makes it difficult to rank them accordingly, however, a clear distinction can still be made between the best and worst producers. The relationship of strain performance and gene copy number in *P. pastoris* has already been demonstrated by Schwarzhans et al*.*^45^. These authors^45^ highlight that this relationship is only observed, if the expression cassettes are orientated head to tail. The obtained WGS data supports this statement, as the investigated strains showed this expression cassette conformation (Supplementary Fig. 4). As a result, it can be concluded that the cumulative oxygen transfer rate correlates to strain performance and can be used as ranking criterion.

**Table 1:**
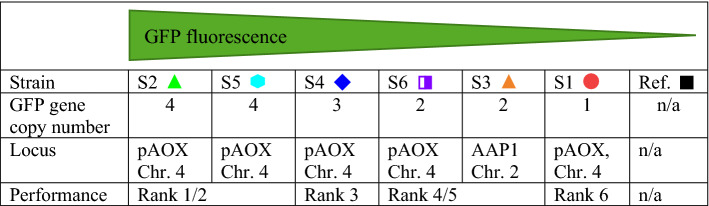
Summary of the Whole Genome Sequencing (WGS) results for the six tested GFP-secreting *P. pastoris* Mut^S^ strains and the reference strain.

The table refers to Figs. 3 and 4 and summarizes the GFP gene copy number present in each tested strain and the integration locus. Strains were ranked according to the gene copy number. An identical gene copy number was evaluated with the same rank. The WGS results, including expression cassette orientation, are shown in Supplementary Fig. 4.

### Demonstrating the applicability of OTR monitoring as a proxy for identifying high protein producers

To demonstrate the potential of online-monitoring the OTR at 96-square well MTP scale, the µTOM device was used to screen a new, larger and uncharacterized clone library. The auto-induction protocol was used to cultivate 45 GFP-secreting *P. pastoris* Mut^S^ strains at 96-square well plate scale. To ensure, that all strains were in the same growth phase prior to inoculation of the screening experiment, cells were grown in a glycerol FeedPlate®. The benefits of this procedure were demonstrated by Keil et al*.*^46^. It was shown that pre-cultures under fed-batch conditions increase the reliability of the screening by synchronizing the cultures of the different strains. This ensures that the initial biomass concentration is comparable for all strains at the beginning of the main culture. As the height of the obtained OTR during phase III is the screening criterion, it is important that all tested strains grow up to a comparable biomass concentration before they are fully induced in growth phase III. Otherwise, the height of the plateau respective of the slope of the cumulative oxygen transfer would be affected by the amount of biomass and not only by the metabolic burden (demonstrated in Fig. 2).

**Figure 6:**
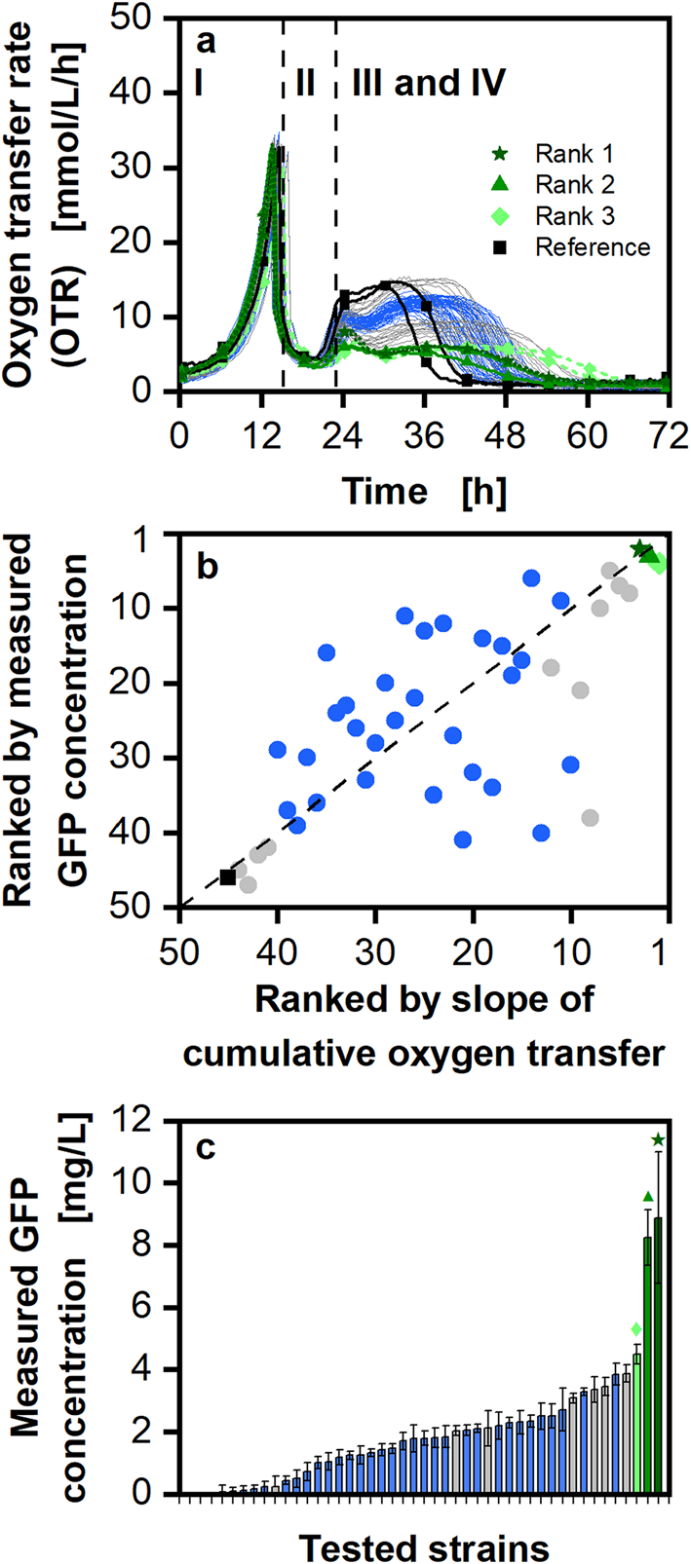
Cultivation of a larger clone library (45 strains) of GFP secreting *P. pastoris* Mut^S^ strains in a 96-square well plate. (**a**) The OTR is depicted for a cultivation with 10 g/L glycerol and 2% (v/v) of methanol, added at the beginning of the cultivation, to obtain auto-induction conditions. For clarity, only every 20th data point is displayed. Cells were cultured at 30 °C in mineral Syn6 medium with 200 mM MES buffer (pH = 6.0). Cultivation conditions: 96-square well plate, 0.6 mL filling volume per well, 350 rpm shaking frequency. 50 mm shaking diameter, initial optical density (OD_600nm_): 0.8. Each cultivation was performed in duplicates (N = 2) and both data sets are displayed. (**b**) The different strains were ranked by the GFP concentration measured at the end of the cultivation (y-axis). The highest GFP fluorescence results in rank 1 (best) and gradually decreases to 45 according to the obtained GFP value. On the x-axis, the slope of the cumulative oxygen transfer (see Supplementary Table 3) was used as ranking criterion. Here, the lowest slope is ranked as 1 (best). If the data points lie on the angular bisector, the ranking result is considered identical for both methods. (**c**) The end point measurements of GFP fluorescence is depicted as a bar graph for the individual strains (measured in four replicates, N = 4). In order to highlight strains of special interest, the same color scheme is used in all three panels to refer to the same strains. The three best performing strains are shown in green and are also represented by a star, triangle, and a rhombus symbol. The worst producer is displayed by a black square symbol. The strains depicted in blue show a very similar OTR course and, therefore, the individual ranks are not clearly assignable. Data displayed in grey shows clearly identifiable differences in the OTR course.

The monitored OTR for the screening is displayed in Fig. 6a. The three best producers are highlighted with a triangle, a rhombus, and a star in different shades of green. All three strains show a lower and longer OTR plateau during phase III. Black squares mark a poorly GFP-producing strain. This strain shows a high and short plateau during phase III. The respective cumulative oxygen transfer rates are shown in Supplementary Fig. 5. The OTR data of most of the strains lie between 10.4 and 12.8 mmol/L/h during phase III. These strains are highlighted in blue, as they all show a very similar growth behavior. Therefore, these strains cannot be clearly differentiated from each other, and the assigned ranks cannot be distinguished. The reason for this color coding becomes clearer within the parity plot (Fig. 6b). Here, the ranks resulting from the slope of the cumulative oxygen transfer (x-axis) are compared with the ranks resulting from the endpoint GFP measurement (y-axis). For identically ranked strains for both methods, the obtained data points should lie on the angular bisector. It becomes apparent that this is the case for the worst producers (black) and the best producers (green). The medium ranks (blue) in the middle are not well defined. Their final GFP concentration is very similar (Fig. 6c), and, therefore scatter around the angular bisector (Fig. 6b).

From the data shown in Fig. 6, it can be concluded that the method presented in this study is suitable to quickly, and reliably, identify good producers in a larger clone bank. This method only requires the evaluation of the course of the OTR signal over time. Neither the biomass nor the protein concentration need to be determined to find good producers. This method is particularly useful when using cultivation systems, for which online biomass determination is not possible. This is the case for the data obtained from 96-square well plates (Fig. 6), as these plates do not have a transparent bottom, and are therefore not accessible for systems such as the BioLector. Nonetheless, this method should be regarded as a pragmatic method for primary-screening with restricted precision. Following primary screening, a re-screening of a limited number of strains should be applied, to ensure the reproducibility of the observed strain performance. This can also be complemented with protein detection methods and quantification assays.

## Conclusion

The genetic toolsets needed to engineer *P. pastoris* strains secreting recombinant proteins have been vastly developed within the last decades^2,47,48^. As a result, not the generation of strains is anymore the bottleneck in bioprocesses development, but the identification of well-performing clones. Its effort naturally increases with the size of the generated clone library and, therefore, it can be time and cost intensive^6^.

In this study, we demonstrated that online monitoring of the course of the OTR over time at 48- and 96-well plate scale, enabled direct assessment of the ranking of GFP production performance for the investigated *P. pastoris* Mut^S^ strains. The ranking results are available directly after the termination of the experiment, without further subsequent efforts, such as protein assays, protein purification via tags, or quantification of fluorescent fusion proteins. While the OTR was comparable during the initial growth on glycerol (phase I), it strongly differed between the GFP-producing *P. pastoris* strains during the subsequent methanol consumption phase (phase III). In phase III, the used *AOX* promoter is induced and initiates GFP production. In general, the more recombinant protein a strain produces, the more pronounced is its metabolic burden, which is visible in reduced biomass formation and breathing activity^49^. As 96-square well plates usually do not have a transparent bottom, the BioLector technology is not applicable for these plates, meaning that the biomass formation cannot be online-monitored. In this study, it could be shown that the OTR signal is already sufficient to make a meaningful prediction. This demonstrates that online monitoring the OTR during primary screening helps to determine potentially good producers, when no other online signal is accessible. The height and length of the OTR plateau in phase III were correlated to metabolic burden effects of different strength. The new screening-method was first demonstrated for 7 pre-selected GFP-secreting strains with known differences. The obtained results were in good agreement with the final offline-measured GFP concentrations. Furthermore, the gene copy number of the integrated GFP expression cassettes led to the same ranking as the one resulting from the slope of the cumulative oxygen transfer. The applicability was also demonstrated using a larger, uncharacterized clone library of GFP-secreting *P. pastoris* Mut^S^ strains. It was shown that the OTR measurements were sufficient to set up a strain ranking that could reliably identify the top producing strains.

Overall, this study demonstrates a time-effective protocol for primary screening, which allows to accelerate the yet laborious selection of high-producing *P. pastoris* Mut^S^ strains. No offline analysis is required to establish a ranking. The protocol results in a pre-selection of strains, reducing the size of the strain library for further thorough testing. Here, we demonstrate the applicability of the described method for the model protein GFP (26.8 kDa)^50^, which is produced under the control of the p*AOX1* promoter. However, it would be interesting to investigate, whether the same trend in the OTR is observed for other strains, promoters or products of larger size.

## Methods

### Microorganisms and strain engineering

All GFP-producing strains are based on the *P. pastoris* host strain BSYBG11 (Mut^S^), obtained from Bisy GmbH (Hofstaetten a. d. Raab, Austria). In this work the BSYBG11 strain is referred to as “reference” (Ref.). It is used as a reference control, as it does not contain a GFP expression cassette. In all used strains, the alcohol oxidase 1 (*AOX1*) was deleted. Therefore, methanol utilization (Mut) is reduced, compared to wild type strains. Only the Aox2 protein is formed to catalyze methanol metabolization. The resulting phenotype is called Mut^S^, and the “s” stands for “slow”, indicating that methanol is consumed at a slower rate^51^. For the GFP-secreting *P. pastoris* strains, the secretion signal mating factor α (MFα) of *Saccharomyces cerevisiae* was fused to the 5’ end of the GFPmut 3 variant^50^. The expression was under the control of the *AOX1* promoter. The used GFP variant has been shown to decrease in signal over time^50,52^. The expression cassette contained GFP under the expression of the *AOX1* promoter and terminator and was randomly integrated into the genome, following the protocol of Lin-Cerenghino et al*.*^53^. Briefly, *P. pastoris* cells were grown overnight in YPD and reinoculated at the following morning until an OD_600_ of 0.8 was reached. Cells were then resuspended in 9 mL of BEDS solution (10 mM bicine-NaOH, pH 8.3, 3% (v/v) ethylene glycol, 5% (v/v) dimethyl sulfoxide, and 1 M sorbitol) with 1 mL 1 M dithiothreitol (DTT). After a 5 min incubation at 30 °C, cells were centrifuged and resuspended in 1 mL BEDS solution, without DTT. 40 μL aliquots were made and 500 ng of the desired DNA was added. The expression vector was linearized prior to transformation, using the SacI restriction enzyme that cuts 208 bp downstream of the AOX promoter. Cells were incubated for 2 min on ice and electroporated, using the GenePulserXcell™ (BioRad, Hercules, CA, USA). The device was set to a voltage of 1500 V, a capacity of 25 μF, and a resistance of 200 Ω. Electroporation was followed by a recovery step in YPD, supplemented with 1 M sorbitol for 2 h at 30 °C. The recovered cells were plated on pre-warmed selection plates containing 1 M sorbitol and 200 µg/mL Zeocin. The strains were sent for WGS using the Oxford Nanopore Technologies’ (ONT) MinION sequencer at CeBiTec, Bielefeld.

### Media and solutions

All chemicals applied for media preparation were of analytical grade and purchased from Carl Roth GmbH (Karlsruhe, Germany), if not stated otherwise.

For the experiments, the microorganisms were grown in mineral Syn6-MES medium. Syn6-MES medium had the following composition^54^: Glycerol was prepared as 500 g/L stock solution and autoclaved separately. The basis Syn6 medium consists of 1.0 g/L KH_2_PO_4_, 7.66 g/L (NH_4_)_2_SO_4_, 3.3 g/L KCl, 3.0 g/L MgSO_4_ × 7H_2_O, 0.3 g/L NaCl, 27.3 g/L 2-(N-morpholino)-ethanesulfonic acid, 4-morpholineethanesulfonic acid (MES). All medium components were dissolved and the pH was adjusted to 6.0 with 1 M NaOH. The basic medium solution was sterilized via autoclaving (121 °C for 20 min). To obtain 1 L Syn6 medium, 940 mL basic medium are supplemented with 10 mL of 100 g/L CaCl_2_ (autoclaved), 10 mL of a 100 × micro-elements stock solution, 10 mL of a 100 × vitamin stock solution, 10 mL of a 100 × trace-elements solution, and the residual 20 mL are used to add a stock solution of the desired carbon source. The stock solutions had the following compositions: Micro-element stock solution: 6.65 g/L EDTA (ethylenediamine tetraacetic acid disodiumsulfat), 6.65 g/L (NH_4_)_2_Fe(SO_4_)_2_ × 6H_2_O, 0.55 g/L CuSO_4_ × 5H_2_O, 2 g/L ZnSO_4_ × 7H_2_O and 2.65 g/L MnSO_4_ × H_2_O. Vitamin stock solution: 0.04 g/L d-biotin and 13.35 g/L thiamine chloride. The d-biotin was dissolved in 10 mL of a (1:1) mixture of 2-propanol and deionized water. Thiamin chloride was dissolved separately in 90 mL deionized water. Afterwards, the two solutions were mixed. Trace element stock solution: 0.065 g/L NiSO_4_ × 6H_2_O, 0.065 g/L CoCl_2_ × 6H_2_O, 0.065 g/L boric acid, 0.065 g/L KI, and 0.065 g/L Na_2_MoO_4_ × 2H_2_O. All stock solutions were filter sterilized.

### Pre-cultures

Pre-cultures were grown in Syn6-MES medium using 10 g/L glycerol as carbon source. Cultivations were performed in 250 mL shake flasks at 30 °C with a shaking frequency of 300 rpm at a shaking diameter of 50 mm for 10–14 h. The liquid culture volume was 10 mL and 125 µL of a cryo-stock was added for inoculation. For the screening of 45 different strains (Fig. 6) the colonies were picked and suspended in individual wells of a glycerol FeedPlate® (Part number: SMFP12002, Kuhner Shaker GmbH, Herzogenrath, Germany). Each well contained 600 µL of Syn6-MES medium (2 × concentrated) without any carbon source. The plates were incubated at 30 °C with a shaking frequency of 350 rpm at a shaking diameter of 50 mm for 48 h.

### MTP cultivations

For cultivations at 48-well MTP scale, an in house built µRAMOS device, in combination with the BioLector technology, was utilized^32,33^. This devices enables online monitoring of the OTR within each individual well. The MTP was sealed with a pierced polyolefin sealing foil (HJ-Bioanalytik GmbH, Erkelenz, Germany) to reduce evaporation and prevent cross contamination. The µRAMOS/BioLector cultivations were conducted in round, 48-well, deep-well microtiterplates (Beckman Coulter GmbH) with a filling volume of 0.8 mL, a shaking frequency of 1000 rpm, a shaking diameter of 3 mm, at 30 °C and a relative humidity higher than 80%. The cell dry weight was calculated using a one-point calibration, where the final cell-dry weight was measured offline and correlated to the online measured backscatter signal. The number of replicates is indicated in the caption of the respective experiment.

For cultivations at 96-well MTP scale, a micro‐scale Transfer rate Online Measurement device (µTOM) was used^34^. Cultivations were conducted in 96-deepwell MTP (riplate SW, 2.5 ml square deepwell plate; HJ‐Bioanalytik GmbH, Erkelenz, Germany), sealed with a gas‐permeable sealing film (AeraSeal™-film, Sigma Aldrich, MA, USA). The filling volume of each well was 0.6 mL, operated at a shaking frequency of 350 rpm, a shaking diameter of 3 mm, at 30 °C and a relative humidity higher than 80%. Initial OD and number of replicates are stated in the caption of the corresponding experiment. All cultivations for protein production were conducted using an auto-induction procedure. The method is described in detail by Wollborn et al*.*^31^. It consists of adding 2% v/v methanol to the cultivation medium at the beginning. As a result, gene expression is induced automatically as soon as the repressing glycerol is consumed, and the cells switch their metabolism towards methanol utilization.

### Methanol detection via HPLC

Methanol was determined by High Performance Liquid Chromatography (HPLC). The HPLC device was equipped with an organic acid resin: pre-column (40 × 8 mm) and an organic acid resin column (250 × 8 mm). Prior to the measurement, samples were centrifuged (10 min at 4000 rpm), filter-sterilized (0.2 μm filter) and stored at 4 °C. 1 mM sulphuric acid was used as eluent. The flow rate was set to 0.8 mL/min at a constant temperature of 40 °C.

### Determination of GFP fluorescence

For GFP offline measurements, a Multi-Detection Microplate Reader (Synergy 4, BioTek, VT, USA) was used. Samples were centrifuged (10 min, 4000 rpm) and 100 μL of cell-free culture supernatant were transferred to a black 96-well flat bottom plate. From each biological sample, 2 technical replicates were measured. Medium, which was used for the corresponding cultivation, was used as a reference blank. The excitation wavelength was set to 488 nm and the emission wavelength was 520 nm. GFP formation was also measured online in the µRAMOS/BioLector system, with an excitation wavelength set to 488 nm and the emission wavelength set to 520 nm. To estimate the concentration of functional GFP, a one-point calibration was performed. Therefore, a GFP standard of known concentration was measured offline and correlated to the online measured GFP signal at the end of the cultivation. The used standard was a full-length GFP with N-terminal His-tag produced in *E. coli* and purified using Ni–NTA agarose. The guaranteed purity was > 70% (EMD Millipore, Temecula, USA).

## Supplementary Information

Supplementary Information.

## Data Availability

The datasets generated during and/or analysed during the current study are available from the corresponding author on reasonable request.
