# Supplementary material for: Predicting high recombinant protein producer strains of Pichia pastoris MutS using the oxygen transfer rate as an indicator of metabolic burden
Source: Sci Rep. 2022 Jul 2;12:11225. doi: 10.1038/s41598-022-15086-w (PMC9250517; doi:10.1038/s41598-022-15086-w)
Supplement: Supplementary file 1 — Supplementary Information. [file 41598_2022_15086_MOESM1_ESM.pdf]

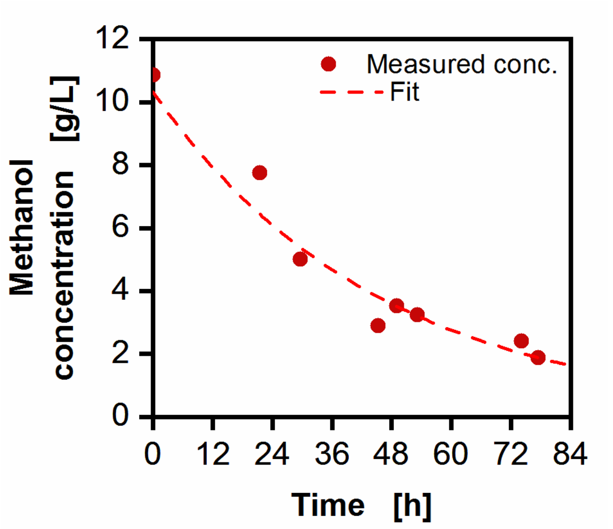

Supplementary Figure 1. Determination of the methanol evaporation rate from 96-square well plates. A concentration of 2% v/v methanol in mineral Syn6 medium with 200 mM MES buffer (pH = 6.0), was added to a 96-square well MTP, 0.6 mL per well. The plate was incubated at 30 °C, 350 rpm and 50 mm shaking diameter. Samples for HPLC analysis were collected over time.

Equation (1)

$$y = 10.32 \cdot e^{0.022 \cdot x}$$
$$R^2 = 0.958$$

Supplementary Table 1. Comparison of theoretical oxygen demand and measured oxygen consumption for the *P. pastoris* Mut^{S} reference strain during growth on methanol. The calculation is based on the data shown in Figure 2, where the duration of the methanol consumption phase is highlighted. The methanol concentration that was available to the cells for the different tested conditions was estimated by considering the methanol evaporation rate, based on the time when phase III ends (Supplementary Figure 1 and Equation 1). To calculate the theoretically required oxygen amount for methanol metabolization, a simplified assumption of biological combustion was made (Equation 2). Row 6 shows the experimentally measured oxygen consumption (Fig. 2) during phase II and III.

**Condition (a)** Glycerol concentration [g/L]	1.5 % v/v Methanol concentration [g/L]	Duration of methanol consumption (Phase III, Fig. 1a) [h]	Residual methanol concentration for cell free wells (evaporation considered) [g/L]	Required oxygen for combustion [mmol/L]	Measured oxygen consumption during growth on methanol [mmol/L]	Deviation between “required” and “measured” oxygen [%]
15	11.88	16.5 - 40	5.22	244.4	266.2 ± 39.6	8.2
10	11.88	14.5 - 45	4.68	219.1	263.2 ± 35.1	16
5	11.88	12.5 - 50	4.20	196.6	235.3 ± 34.5	16
**Condition (b)** Glycerol concentration [g/L]	2 % v/v Methanol concentration [g/L]	End of methanol consumption (Phase III, Fig. 1b) [h]	Residual methanol concentration for cell free wells (evaporation considered) [g/L]	Required oxygen for combustion [mmol/L]	Measured oxygen consumption during growth on methanol [mmol/L]	Deviation between “required” and “measured” oxygen [%]
15	15.84	16.5 - 48	5.96	279.0	338.6 ± 31.1	17.6
10	15.84	14.5 - 48	5.96	279.0	325.2 ± 10.1	14.2
5	15.84	12.5 - 55	5.11	239.2	297.6 ± 17.7	19.6

Equation (2) $$C_1H_4O_1 + 1.5\ O_2 \rightarrow 1\ CO_2 + 2\ H_2O$$

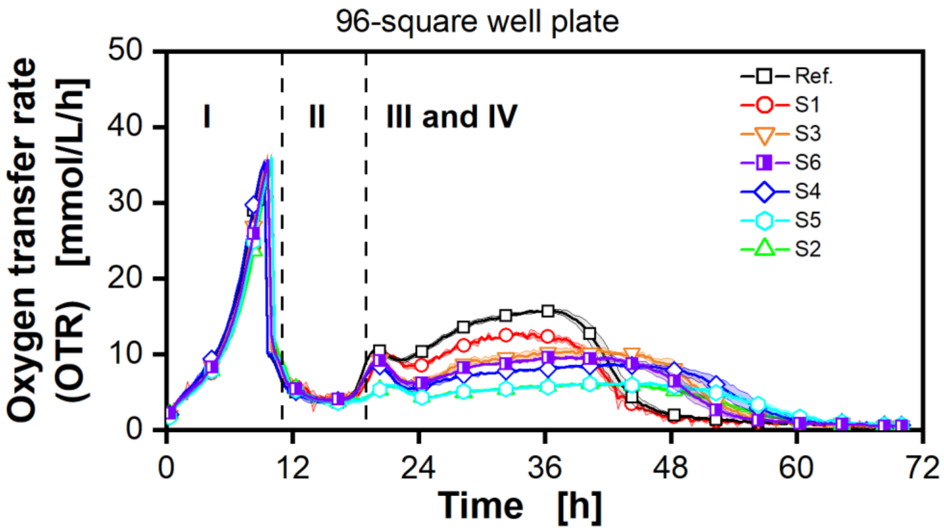

Supplementary Figure 2. Cultivation of a clone library with GFP secreting *P. pastoris* Mut^S strains in a 96-square well microtiter plate with online monitoring of the oxygen transfer rate (OTR). Results are depicted for a cultivation with 10 g/L glycerol and 2% (v/v) of methanol added at the beginning of the cultivation, to obtain auto-induction conditions. For clarity, only every 12th data point is displayed. The four cultivation phases (I-IV) are indicated by vertical dashed lines, as in the previously shown data set (Fig. 3). Cells were cultured at 30°C in mineral Syn6 medium with 200 mM MES buffer (pH = 6.0). Cultivation conditions: 0.6 mL filling volume per well, 350 rpm shaking frequency. 50 mm shaking diameter, initial optical density (OD_{600nm}): 0.8. Each cultivation was performed in six replicates (N=6). Shadows symbolize the standard deviation, which in some cases is so small, that it can't be seen. Strains S2 and S5 perform almost identical and therefore are plotted on top of each other, thus they are difficult to distinguish.

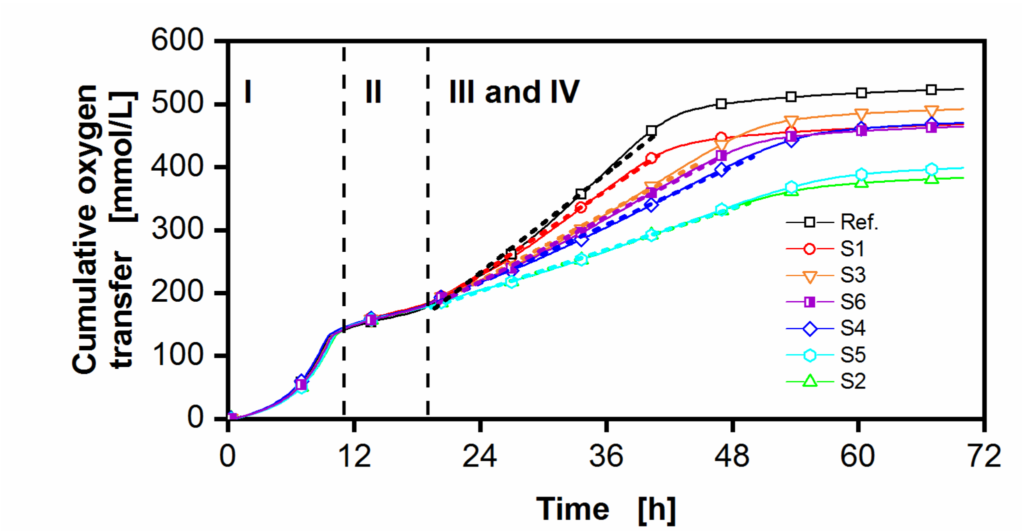

Supplementary Figure 3. Cumulative oxygen transfer data of a clone library with GFP secreting *P. pastoris* Mut^S strains cultivated in a 96-square well microtiter plate. The integral of OTR is depicted for the cultivation shown in Supplementary Fig. 2, with 10 g/L glycerol and 2% (v/v) of methanol added at the beginning of the cultivation, to obtain auto-induction conditions. For clarity, only every 20th data point is displayed. The four cultivation phases (I-IV) are indicated as in the previously shown data set (Fig. 3 and 4). Cells were cultured at 30°C in mineral Syn6 medium with 200 mM MES buffer (pH = 6.0). Cultivation conditions: 0.6 mL filling volume per well, 350 rpm shaking frequency. 50 mm shaking diameter, initial optical density (OD_{600nm}): 0.8. Each cultivation was performed in six replicates (N=6).

Supplementary Table 2. Summary of the slopes obtained in Fig. 4 and in Supplementary Fig. 3 from the cumulative oxygen transfer. The slopes were obtained by a linear regression of phase III of cultivations in 48-round well microtiter plates and in 96-square well microtiter plates. Order of strains in (**a**) according to Figure legend of Figure 3. In part (**b**) of the table the position of S6 and S3 is inverted.

	**a)**		**b)**	
**Tested *P. pastoris* Mut^S strain**	**Slope obtained in 48-round well plate [mmol/L/h]**	**Ranking result in 48-round well plate**	**Slope obtained in 96-square well plate [mmol/L/h]**	**Ranking result in 96-square well plate**
Reference	17.2	7	14.6	7
S1	14.7	6	12	6
S6	11	5	8.8	4
S3	10	4	9.3	5
S4	8.8	3	7.6	3
S5	7.2	2	5.4	2
S2	6.7	1	5.4	1

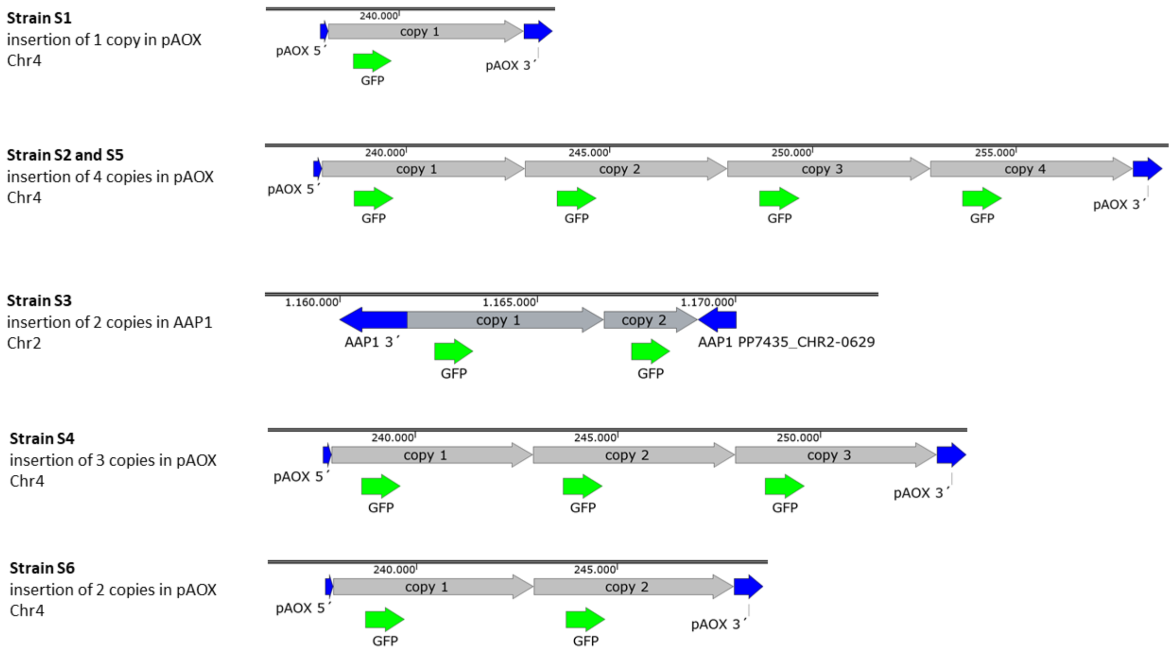

Supplementary Figure 4. Whole Genome Sequencing results. The orientation, locus and number of integrated GFP expressing cassettes is displayed for the tested strain library.

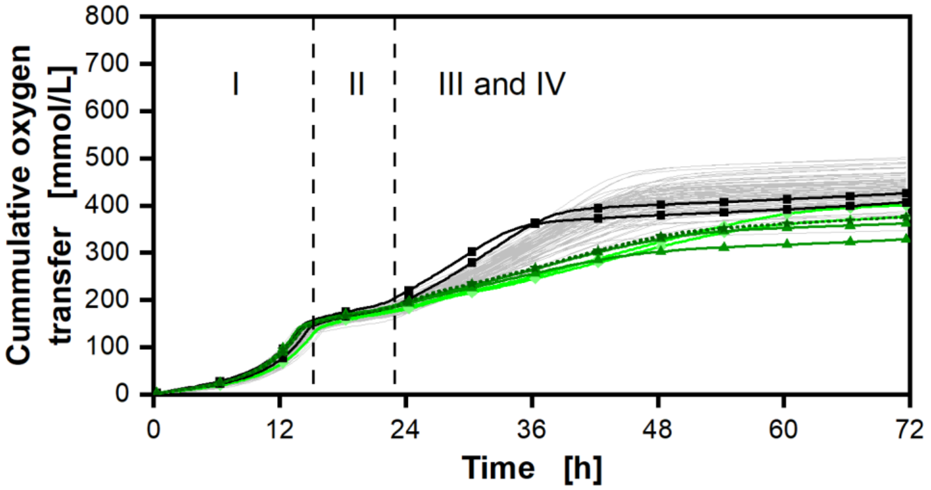

Supplementary Figure 5. Cumulative oxygen transfer data of a larger clone library (45 strains) of GFP secreting *P. pastoris* Mut^S strains in a 96-square well plate. The integral of the OTR shown in Figure 6a is depicted for the cultivation with 10 g/L glycerol and 2% (v/v) of methanol added at the beginning of the cultivation, to obtain auto-induction conditions. For clarity, only every 18th data point is displayed. The four cultivation phases (I-IV) are separated by vertical dashed lines as in Fig. 1. Cells were cultured at 30°C in mineral Syn6 medium with 200 mM MES buffer (pH = 6.0). Cultivation conditions: 0.6 mL filling volume per well, 350 rpm shaking frequency. 50 mm shaking diameter, initial optical density (OD_{600nm}): 0.8. Each strain was cultivated in duplicates.

Supplementary Table 3. Summary of the slopes obtained in Supplementary Fig. 5 from the cumulative oxygen transfer. The slopes were obtained by a linear regression of phase III of cultivations in 96-square well microtiter plates. The best producers (Rank 1 to 3) and the reference are marked with the same symbols as in Figure 6 and Supplementary Figure 5.

**Strain No.**	**Slope of cumulative oxygen transfer**	**Strain No.**	**Slope of cumulative oxygen transfer**	**Strain No.**	**Slope of cumulative oxygen transfer**	**Strain No.**	**Slope of cumulative oxygen transfer**
1	7.58	27	11.20	45	9.75	56	10.08
1	7.72	29	8.74	45	10.14	57	11.93
3	9.41	29	9.40	46	9.48	57	11.58
3	9.77	30	9.64	46	9.50	58	9.49
8	9.09	30	10.26	47	9.36	58	9.85
8	9.87	31	9.43	47	10.29	59	8.61
11	9.62	31	10.38	48	9.47	59	9.80
11	10.14	32	9.14	48	10.28	60	9.44
13	9.28	32	9.85	49	9.54	60	10.38
13	9.99	35	10.09	49	9.98	61	9.62
14	5.47 ◆	35	10.19	50	9.80	61	10.10
14	5.24 ◆	36	10.15	50	9.68	62 ■	13.28
16	7.08	36	9.96	51	8.79	62 ■	12.53
16	7.09	40	9.63	51	9.13	63 ▲	5.25
18	7.50	40	9.86	52	6.14	63 ▲	5.66
18	8.24	41	7.20	52	6.65	64	9.87
20	11.82	41	7.71	53	9.13	64	10.28
20	12.17	42	6.60	53	9.88	65	9.31
23	9.38	42	7.21	54	9.70	65	10.56
23	9.49	43	9.69	54	10.18	66 ★	5.69
26	11.32	43	10.71	55	9.48	66 ★	5.84
26	11.73	44	9.70	55	10.19	56	10.08
27	10.94	44	9.92	56	10.12	57	11.93
